# Influence of Light-Curing Mode on the Erosion Preventive Effect of Three Different Resin-Based Surface Sealants

**DOI:** 10.1155/2012/874359

**Published:** 2012-09-06

**Authors:** Florian J. Wegehaupt, Tobias T. Tauböck, Beatrice Sener, Thomas Attin

**Affiliations:** Clinic for Preventive Dentistry, Periodontology and Cariology, University of Zurich, Plattenstrasse 11, 8032 Zürich, Switzerland

## Abstract

*Objectives*. To investigate if reducing the light-curing time (while maintaining similar energy density) of resin-based surface sealants influences their erosion-preventive potential and mechanical stability after thermomechanical loading. *Methods*. Dentine samples were treated as follows: group 1—untreated, groups 2–4—Seal&Protect, groups 5–7—experimental sealer, and groups 8–10—Syntac Classic system. Groups 2, 5 and 8 were light-cured for 10 s (1000 mW/cm^2^), groups 3, 6 and 9 for 7 s (1400 mW/cm^2^), and groups 4, 7, and 10 for 3 s (3200 mW/cm^2^). After water storage (7 d), first measurement was performed to evaluate baseline permeability of the sealants. After a thermomechanical loading (5000 cycles, 50/5°C, 12000 brushing strokes) a second evaluation of permeability was conducted (measurement 2). Permeability was tested by storing the samples in HCl (pH 2.3; 24 h) and measuring the dentine calcium release by atomic absorption spectroscopy. *Results*. For the first and second measurements, no influence of light-exposure time on permeability was observed (ANOVA: *P* > 0.05). No significant difference in the stability of the respective sealants was observed when light-cured for different durations. *Conclusion*. Shortening the light-curing time, while maintaining energy density constant, has no influence on permeability and stability of the investigated sealants.

## 1. Introduction

In industrialised countries a significant decrease of the caries prevalence has been observed during the last decades [1]. In the last few years, studies reporting a high prevalence (up to 32%) of erosion in certain patient groups (children or patients with gastrooesophageal reflux disease) have been published [2, 3]. Despite these studies, it is not clear if the prevalence of dental erosion is truly increasing or if only the increasing awareness about erosion results in a more precise diagnosis and more pronounced perception erroneously interpreted as an increased prevalence.

In order to prevent erosive enamel and/or dentine wear, different preventive approaches such as strengthening the chemical resistance of the dental hard tissues [4] or rehardening of erosively softened enamel or dentine have been discussed. Many of these preventive approaches based on the application of different fluoride compounds and formulations, namely, amine fluoride, sodium fluoride, sodium monofluorophosphate, titanium tetrafluoride, and stannous fluoride on enamel or dentine. Such preventive approaches act as therapeutics, which increase the acid resistance of the so treated dental hard tissues *in situ* and *in vitro* [5, 6].

Further, the erosive loss of dental hard tissue could be prevented by hampering the contact of the erosion causing substrates with the dental hard tissues by means of a mechanical barrier on the enamel or dentine surfaces [7]. In 2000, Brunton et al. [8] suggested a coating of dentine with a resin-based dentine adhesive to prevent erosive/abrasive wear. In a recent study [9], Seal&Protect (DENTSPLY DeTrey GmbH, Konstanz, Germany) showed a good long-term protective effect against enamel erosive wear. As Seal&Protect contains triclosan, for which possible negative side effects like induction of antibiotic resistances and accumulation in human milk were reported [10, 11], an experimental sealant without triclosan should be also tested in the present study. Furthermore, the Syntac Classis system should be tested as a representative of adhesive systems like suggested by Brunton et al. [8].

According to the manufactures' instructions these sealants must be light-cured at least two times for 10 s per tooth surface to be sealed, when using common polymerisation device settings (approximately 1000 mW/cm^2^ output intensity). This fact might be regarded as disadvantageous, since it might be too time consuming, when multiple tooth surfaces have to be sealed.

Therefore, the aim of the present study was to investigate whether shortening the light-curing time has an influence on the erosion prevention potential of surface sealants.

As shortening the light-curing time may result in an unfavourable lower degree of polymer conversion [12] with inferior mechanical properties [13, 14] and higher cytotoxicity [15] of resin-based materials, it might be of interest to compensate the shorter light-curing time by increasing the light intensity. It is known that increasing the light intensity results in an increased degree of conversion [16], however also higher shrinkage stress was observed when resin-based materials were light-cured with higher intensities [17, 18]. This higher shrinkage stress might provoke an insufficient sealing of the dentine by the so cured surface sealants (shorter curing-time but higher curing intensity). Furthermore, it has to be taken in to consideration that increasing the light intensity may cause an increase of temperature in the pulp chamber [19].

The primary hypothesis of the present study was that shortening the light-curing time while simultaneously increasing light intensity results in a reduced erosion-preventing efficacy of the surface sealants. The secondary hypothesis was that surface sealants provide an erosion preventing effect and that effect will last under thermomechanical loading.

## 2. Materials and Methods

### 2.1. Sample Preparation

For the study, 120 dentine samples were prepared from freshly extracted bovine (age under 36 months) lower incisors. The teeth were sectioned at the cementum-enamel junction with a water-cooled diamond-coated disc. The pulp tissue was removed from the roots with endodontic files.

From the distal and mesial surface of each root, samples were gained with a trephine mill. The inner diameter of the drill amounted to 3 mm. The dentine cylinders were embedded in acrylic resin (Palavit G, Kulzer, Wehrheim, Germany) in metal moulds with an inner diameter of 6 mm. The dentine surface was ground with abrasive paper (800, 1000, 1200, 2400, and 4000 grit; Water Proof Silicon Carbide Paper, Streuers, Erkrat, Germany). In this grinding step, the cementum was removed, which was additionally checked with a stereomicroscope (40x).

**Figure 1:**
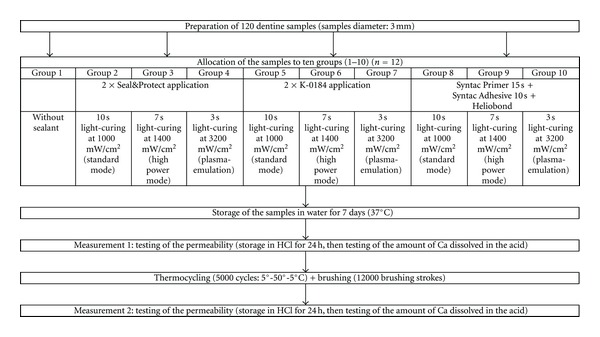
Sample allocation and experimental procedure.

After sample preparation, samples were randomly allocated to ten groups (1–10) with 12 samples per group. Sample allocation and experimental procedure is shown in a flow chart (Figure 1).

### 2.2. Treatment of the Samples

The samples in group 1 remained untreated and served as a control group.

Samples of the groups 2–4 were treated with Seal&Protect (pH 2.5–3; DENTSPLY DeTrey GmbH, Konstanz, Germany), while the samples in groups 5–7 were treated with K-0184 (pH 2.5–3; experimental sealer; DENTSPLY DeTrey GmbH, Konstanz, Germany). The respective sealants were applied on the dentine surface and left undisturbed for 20 s. After these 20 s, the remaining solvent was removed with an air syringe, and the sealant was light-cured. A second layer of sealant was applied, the solvent evaporated with an air syringe and light-cured again. Samples in groups 8–10 were treated with Syntac Primer (pH: 1.6; Ivoclar Vivadent, Schaan, Liechtenstein) for 15 s (gently rub in), excess was dispersed and thoroughly air-dried. Syntac Adhesive (Ivoclar Vivadent, Schaan, Liechtenstein) was applied, left for 10 s, and again thoroughly dried with an air syringe. Finally, Heliobond (Ivoclar Vivadent, Schaan, Liechtenstein) was applied, blown to a thin layer and light-cured. The composition of the used materials is given in Table 1.

Light curing was performed with the VALO LED light-curing device (Ultradent Products, South Jordan, USA). In groups 2, 5, and 8 light curing was performed at standard mode (1000 mW/cm^2^) for 10 s (=10 J/cm^2^), in groups 3, 6, and 9 at high power mode (1400 mW/cm^2^) for 7 s (=9.8 J/cm^2^) and in groups 4, 7, and 10 at plasma-emulation mode (3200 mW/cm^2^) for 3 s (=9.6 J/cm^2^). The light curing units were checked for consistency prior to curing using a radiometer (Optilux Radiometer, SDS Kerr; Orange, CA, USA). Holding the samples with a forceps and resting the light output window on the forceps guaranteed a constant distance between light-curing tip and samples surface of 0.5 cm.

### 2.3. Analysis of Sealer Permeability and Stability

After one-week storage in water (37°C, distilled water), the first measurement was performed to evaluate baseline permeability of the surface sealants. For this, samples were stored in hydrochloric acid for 24 h (pH 2.3; 4.5 mmol/L). Each sample was stored in a separate Eppendorf Tube (Eppendorf International, Hamburg, Germany) with 1 mL of HCl under constant motion.

For testing permeability of the sealants, the amount of dentine calcium dissolved in the HCl was measured. For this determination, 500 *μ*L of the HCl was mixed with the same amount of water and strontium chloride (0.25%). Strontium chloride was added to mask the phosphate dissolved in the acid that might otherwise falsify the following measurement of calcium by atomic absorption spectroscopy (2380 Atomic Absorption Spectrophotometer, Perkin-Elmer, Schwerzenbach, Switzerland). Measurement was performed at 422.7 nm.

To test stability of the surface sealants, the samples were subjected to the following thermomechanical loading: 5000 cycles of changing the surrounding water temperature every 120 s from 5°C to 50°C and 12000 brushing strokes (BS) with toothpaste slurry in an automatic brushing machine applying reciprocating linear motion to the toothbrushes (ParoM43, Esro AG, Thalwil, Zürich, Switzerland). The brushing machine was adjusted to a constant brushing frequency of 120 strokes per minute and a constant brushing load of 2.5 N. The toothpaste slurry was prepared by mixing 300 mL artificial saliva (composition given by Klimek et al., 1982) [20] and 100 mL toothpaste (elmex, Gaba, Münchenstein, Switzerland; 1400 ppm, amine fluoride, RDA: 75). After each 500 brushing strokes, the slurry was renewed.

After this thermomechanical loading, the permeability of the sealant was again tested as described above (second measurement). Before storing the samples in the HCl, the samples were thoroughly cleaned with deionised water.

## 3. Statistical Methods

Data were coded in EXCEL and analyzed with SPSS Version 16.

Descriptive statistics such as mean and standard deviation of calcium released (SD) were computed for each group (1–10) at each measurement (measurement 1 and 2) and for the cumulative calcium release (measurement 1 plus 2) and was interpreted as the permeability of the materials.

Furthermore, the difference in the calcium release was calculated (calcium release in measurement 1; calcium release of the respective sample in measurement 2 = Δ calcium release). This difference was interpreted as stability of the surface sealants. Lower values represent a lower stability of the surface sealant with a higher susceptibility to thermomechanical and erosive wear.

The assumption of normal distribution of errors was checked, using Kolmogorov-Smirnov test.

Statistical analysis was performed using ANOVA followed by Scheffé post hoc tests in order to investigate the differences in the amount of calcium dissolved in the experimental groups 2–10 and to compare the stability of the surface sealants (Δ calcium release). The calcium release in the treated groups (2–10) was compared with that of the unsealed control group (1) by ANOVA followed by Dunnett *t*-test. 

Results of the statistical analysis with *P* values < 5% were interpreted as statistically significant.

## 4. Results

### 4.1. Sealer Permeability

Calcium release during the first and second measurements and the cumulative calcium release in the groups 1–10 are presented in Table 2.

At the first measurement, ANOVA revealed no significant influence of light exposure time on the calcium release (*P* = 0.704).

At the first measurement, the highest calcium release was observed for the untreated control group (103.4 ± 30.3 *μ*g). The calcium release in the samples sealed with Seal&Protect (groups 2, 3, and 4) and K-0184 (groups 5, 6, and 7) were significantly lower compared to the calcium release of groups 8, 9, and 10 (Syntac Classic system) at the corresponding light exposure times (*P* < 0.05, resp.).

Also at the second measurement, no significant influence of the light-curing time on the calcium release could be observed (ANOVA; *P* = 0.205).

At this timepoint, calcium release in the sealed samples was either not significantly different (groups 2, 3, 5, 7–10) or significantly higher (group 4: Seal&Protect 3 s) compared to the unsealed control group (75.3 ± 29.9 *μ*g). Only group 6 (K-0184 7 s) showed a significantly lower calcium release.

Moreover, the data showed that cumulative calcium release was not significantly influenced by the different light exposure times (ANOVA; *P* = 0.660).

The cumulative calcium release in all groups treated with surface sealants was significantly lower compared to the cumulative calcium release in the unsealed control group (*P* < 0.05, resp.).

### 4.2. Stability of Surface Sealants

**Figure 2:**
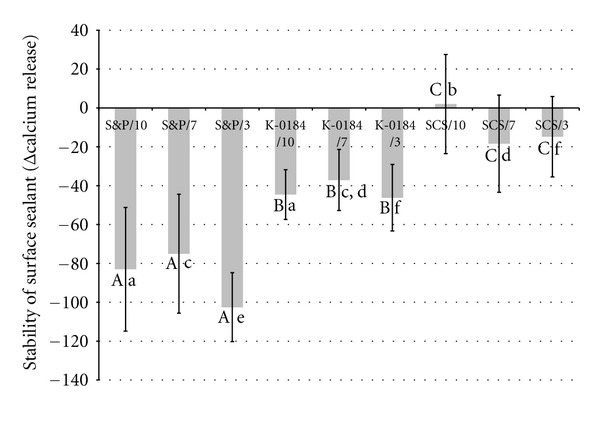
Stability (mean Δ calcium release (*μ*g) ± SD) (*v* = 500 *μ*L) of the respective surface sealants cured with different light exposure times. (S&P = Seal&Protect, K-0184 = experimental sealer and SCS = Syntac Classis system; 10 = 10 s light-curing time, 7 = 7 s light-curing time, etc.) Lower values indicate lower stability of the surface sealants. Within the same material, values for different light-curing durations, which are not statistically significantly different, are marked with identical uppercase letters. Comparisons between values within the same light-curing duration that are not significantly different are marked with same lower case letters.

The stability of the respective surface sealants applied with different light exposure times is presented in Figure 2.

For the stability of the surface sealants, a significant influence of the light exposure times could be observed (ANOVA; *P* = 0.0404). However, the respective post hoc tests showed no significant differences in the stability of the respective surface sealants when light cured for different durations (*P* > 0.05, resp.).

Within the 10 s light exposure time, the significantly highest stability was observed for the Syntac Classis system with no significant difference between Seal&Protect and K-0184. At 7 s light-curing duration, the stabilities of Seal&Protect and K-0184 were not significantly different (*P* = 0.101). Also the stabilities of the Syntac Classis system and K-0184 were not significantly different (*P* = 0.932). When light-cured for 3 s, the significantly lowest stability was observed for Seal&Protect.

## 5. Discussion

For the present study, samples were prepared from bovine dentine. Bovine teeth have the advantage that they are easy to obtain and that between two and six teeth can be harvested from one animal, while this number of teeth can rarely be gained from one human subject. Additionally, bovine teeth used for studies mostly stem from cattle raised in a comparable environment, with similar forage. Further, these teeth do not have a history of caries and/or fluoridation measures as many human teeth have, which might influence erosive demineralization or adhesion of applied surface sealants. Previous studies have proven bovine dentine to be a suitable alternative for human dentine in *in vitro* studies with regards to permeability characteristics [21] and adhesion tests [22]. In addition, human and bovine dentine does not perform differently under the same *in vitro* erosion/abrasion conditions [23]. Although it is favourable to use human dentine, it seems to be acceptable to substitute human with bovine dentine especially when the values of the respective test groups are compared with each other and with the untreated controls of the same study (relative values).

A limitation of the present study might be that a smear layer was present on the dentine surfaces after the sample preparation. Under clinical conditions no such smear layer would be found. This smear layer might have an influence on the interaction of the sealants with the dentine during application, resulting in a poorer quality of the bonding of the sealants to the dentine. As the surface sealants used have an acidic pH (manufacturers information: Seal&Protect and K-0184: pH 2.5–3 and Syntac Primer: 1.6), it might be assumed that they are all able to remove or incorporate the minerals of the smear layer. Furthermore, it might be assumed that due to its lower pH, Syntac Primer is able to modify and/or remove the smear layer and to demineralise the dentine during bonding better than Seal&Protect and K-0184. However, as all sealants used provided a good protective effect, even under these disadvantageous conditions, it might be assumed that under clinical conditions at least the same protective effect and stability of the sealants might be observed. It has to be taken in consideration that the performance (antierosive effect) of the Syntac Classic system is not only determined by the pH of Syntac Primer but also by the performance of the later applied Syntac Adhesive and Heliobond.

Simulation of the erosive attack was performed with pure hydrochloric acid. Hydrochloric acid was selected for use, as it is the acidic compound found in stomach content [24]. However, beside hydrochloric acid, the gastric juice also contains various enzymes [24]. One such enzyme, pepsin, is a proteolytic enzyme well known for its ability to degrade collagen [25, 26]. A previous study has shown that the erosive mineral loss from dentine is higher, when the collagen matrix, exposed during the erosive attack, is removed [27]. Taking these findings into consideration, it is imaginable that an admixture of pepsin to the hydrochloric acid results in a higher erosive dentine loss. However, a study by Schlueter et al. (2007) [28] found no influence of pepsin admixture on hydrochloric-acid-induced dentine loss. Nevertheless, another study [29] by the same group showed an intensified dentine erosion progression *in vitro* when pepsin and trypsin were added to the hydrochloric acid. As the results of these studies are inconclusive, in the present study the simulation of the erosive attack was performed with pure hydrochloric acid, as performed in numerous other studies. In the present study, hydrochloric acid with a pH of 2.3 (titratble acidity: 0.05 mL of 0.05 M NaOH for 1 mL HCl) was used to simulate gastric juice. For gastric juice, Bartlett and Coward (2001) [30] found a mean pH of 2.92 (range 1.2–6.78) (in seven gastric acid samples) and mean titratable acidity of 0.68 mL of 0.05 M NaOH (range 0.03–1.64). However, it has to be taken in to consideration that Bartlett and Coward (2001) [30] found a broad range for both the pH and the titratable acidity of the gastric juice, which also covers the pH and titratable acidity of the hydrochloric acid solution used in the present study. Furthermore, we assume that using a higher pH, more like the one of the gastric juice found by Bartlett and Coward (2001) [30], might result in lower amounts of calcium dissolved in the same time periods but should not fundamentally change the findings of the present study.

The primary hypothesis of the present study, which is shortening the light-curing time while simultaneously increasing of the light intensity, results in a worse erosion-preventing efficacy of the surface sealants, has to be rejected. No significant influence of the light-curing duration on the permeability of the respective surface sealants was observed; neither at the initial measurement 1, final measurement 2, nor for the overall performance (cumulative calcium loss) of the surface sealants. It might be hypothesised that the shrinkage stress of the resin-based surfaces sealants, which might be influenced by the increasing light-curing intensity [31], had no negative effect on the permeability of the surface sealants used. This might be explained with the observation that during setting of resin-based materials, shrinkage stress can be compensated by flow of incompletely polymerised compounds [32].

Furthermore, it has to be taken in to consideration that the negative influence of high light intensities on shrinkage stress of resin-based dental materials was found in cavities simulating disadvantageous cavity configuration [18, 33]. In the present study, the material was applied in a flat layer on the dentine surface resulting in one bonded surface only, thus representing a favourable C-factor with low shrinkage stress during polymerisation [34].

The mechanical stability of resin-based materials is influenced by the degree of conversion [13]. Shortening the light-curing time without increasing the light intensity might reduce the degree of conversion of the surface sealants [12], which might then affect their stability. In the present study, no significant differences in the stability values of the respective surface sealants were observed when the surface sealants were light-cured for different durations but using similar energy densities. Nevertheless, it should be clarified in further studies if the polymerisation approaches used in the present study might have an influence on the material properties, such as biocompatibility and degree of conversion, and on temperature changes in the pulp chamber. However, a recent study [19] found the increase in temperature in the pulp chamber directly related to the light intensity and exposure time and recommend that curing devices with high power density (>1200 mW/cm^2^) should only be activated for a short period of time (<15 s). As in the present study the light density over 1200 mW/cm^2^ was used for a maximum of 7 s, it might be assumed that this application mode does not have a negative influence on the pulp under clinical conditions.

The absolute stability values and the existing differences in the stability values of the different materials tested have to be interpreted with caution, due to the method of calculating the stability in the present study (calcium release in measurement 1; calcium release of the respective sample in measurement 2). In the Syntac Classic system group, for which slightly better stability values have been calculated, at the initial measurement (measurement 1), directly after application of the sealants without any loading, the permeability was between 8 and 17 times higher than that of the other sealants. However, at the second measurement, no difference in the permeability of the different surface sealants was observed. Thus, it has to be acknowledged that the superior stability of the Syntac Classic system was due to the fact that the initial permeability was already high, or in other words, that the antierosive effect was poor compared with the other surface sealants used. When looking at the values for the cumulative calcium loss, representing the performance of the products during the whole experimental procedure, it becomes obvious that the protective effect of the Syntac Classic system might be similar or even worse than that of the other sealants.

Beside the commercially available Seal&Protect, the experimental sealant K-0184 was also tested in the present study. The chemical composition of this sealant is similar to Seal&Protect, with the single difference that no triclosan is incorporated in the experimental sealant. As no significant difference in the cumulative calcium release and the stability values between Seal&Protect and the triclosan-free experimental sealant were observed, one might conclude that absence or presence of triclosan in the sealant used has no significant effect on its antierosive effect and stability.

The secondary hypothesis of the present study has to be partially rejected. In the present study the tested surfaces sealants showed a very good protective effect against erosive demineralisation during the first measurement (24 h erosion in hydrochloric acid). For the samples sealed with the tested surface sealants a reduction of the calcium release of 51% (Syntac Classic system light-cured for 10 s) up to 98% (Seal&Protect light-cured for 7 s) was observed. These findings are in accordance with the findings of a recent study [9], investigating the protective effect of surface sealants against erosive enamel wear caused by extrinsic and intrinsic acids under long-term exposition. In that study, a reduction of 81% of the calcium release during a 24 h storage in hydrochloric acid was found after application of Seal&Protect. Furthermore, Seal&Protect was able to provide a protective effect up to 4 days against erosive demineralisation caused by HCl.

At the final measurement (2) of the present study, after the thermomechanical loading, no further differences in the calcium release from the sealed samples, as compared to that of the unsealed control samples, were observed. Due to this finding, it might be concluded that the tested surface sealants are not able to maintain the initially observed antierosive protective effect during the thermomechanical loading. However it has to be taken in consideration that the mechanical loading used here, 12000 brushing strokes (BS), represents approximately 13 to 20 months *in vivo*, following the findings of a recent study by Wiegand and Attin (2011) [35]. They assumed 10–15 brushing strokes per tooth during a single tooth brushing session being adequate to simulate the clinical condition *in vitro*. Assuming tooth brushing twice a day, the 12000 BS of the present study equals 400 to 600 days under clinical conditions.

In 1996, Bartlett et al. [36] found a drop of the oral pH below 5.5 for 0.3% and below pH 6 for 4.4% of the total time during 24-hour pH telemetry in gastrooesophageal reflux patients. This corresponds to an erosion time between 4.3 and 60 min per day, respectively. Taking these findings into account, the here used 24 h erosive attack is equivalent to 24–330 days under clinical conditions. As the erosive attack has been performed twice, this results in a total simulation time of up to 660 days, similar to the duration simulated by the tooth brushing.

## 6. Conclusion

By the findings of the present study it can be concluded that

1. shortening the light exposure times, while maintaining the energy density, has no negative influence on the erosion prevention potential and stability of the surface sealants used;
2. initially (first 24 h erosion) all surface sealants show a good protective effect against erosive demineralisation, with a loss of this protective effect due to the thermomechanical loading used here;
3. further studies are needed to evaluate how long the observed protective effect will last under more *in vivo*-like conditions.

## Figures and Tables

**Table 1:** Composition of the used surface sealants and adhesive system (manufacturer's information).

Product	Composition
Seal&Protect (Dentsply Detrey, Konstanz, Germany)	Di- and trimethacrylate resins, PENTA (dipentaerythritol penta acrylate monophosphate), functionalised amorphous silica, photoinitiators, butylated hydroxytoluene, cetylamine hydrofluoride, triclosan, acetone
K-0184 (Dentsply Detrey, Konstanz, Germany)	Di- and trimethacrylate resins; PENTA (dipentaerythritol penta acrylate monophosphate), functionalised amorphous silica, photoinitiators, butylated hydroxytoluene, cetylamine hydrofluoride, acetone
Syntac Classic system (Ivoclar Vivadent, Schaan, Liechtenstein)	Syntac Primer: triethylene glycol dimethacrylate, polyethylene glycol dimethacrylate, maleic acid and acetone in an aqueous solution Syntac Adhesive: polyethylene glycol dimethacrylate and glutaraldehyde in an aqueous solution Heliobond: Bis-GMA, triethylene glycol dimethacrylate, stabilizers and catalysts

**Table 2:** Sealer permeability.

Group	Material	Light-curing time (s)	Measurement 1	Measurement 2	Cumulative
1	Unsealed control		103.4 (30.3)	75.3 (29.9)	178.7 (50.6)
2	S&P	10	*2.9^a^ (2.2)	85.9^a^ (31.8)	*88.8^a^ (31.9)
3		7	*2.5^c^ (2.5)	77.5^b^ (29.7)	*80.1^b^ (28.9)
4		3	*2.6^e^ (2.1)	*105.1^c^ (20.2)	*107.8^c^ (20.9)
5	K-0184	10	*6.1^a^ (7.6)	50.7^a^ (16.0)	*56.8^a^ (21.4)
6		7	*5.7^c^ (4.4)	*42.8^b^ (15.3)	*48.4^b^ (16.1)
7		3	*2.9^e^ (1.7)	49.1^d^ (17.0)	*52.0^c^ (17.1)
8	SCS	10	*50.2^b^ (26.9)	48.2^a^ (28.7)	*98.3^a^ (49.5)
9		7	*47.1^d^ (27.9)	65.5^b^ (32.3)	*112.6^b^ (55.0)
10		3	*44.0^f^ (29.0)	58.7^d^ (28.0)	*102.7^c^ (53.1)

Calcium release *μ*g (±SD) (*v* = 500 *μ*L) in the different groups (1–10) (S&P: Seal&Protect, K-0184: experimental sealer and SCS: syntac classic system).

Values that are significantly different to the respective untreated controls are marked with*.

Within the same measurement and same material, values for different light-curing durations, were not statistically significantly different.

Comparisons between values within the same measurement and same light-curing duration for different materials that are not significantly different are marked with same lower case letter.
